# High‐Protein Diet Combined With *Lactobacillus acidophilus* Improves Weight Loss, Lipid Metabolism, and Gut Microbiota in Obese Rats

**DOI:** 10.1002/fsn3.72118

**Published:** 2026-07-22

**Authors:** Aicha Sbaihia, Bouasria Benbouziane, Muhammad Waqar, Mohamed Cherif Bentahar, Djilali Benabdelmoumene, Said Dahmouni, Soumia Keddari, Wasim S. M. Qadi, Ahmed Mediani, Temesgen Anjulo Ageru

**Affiliations:** ^1^ Laboratory of Bioeconomics, Food Safety and Health, Faculty of Natural Sciences and Life Abdelhamid Ibn Badis University of Mostaganem Mostaganem Algeria; ^2^ Food Technology and Innovation Research Center of Excellence, School of Agricultural Technology and Food Industry Walailak University Nakhon si thammarat Thailand; ^3^ Laboratory of Applied Animal Physiology, Faculty of Natural Sciences and Life Abdelhamid Ibn Badis University of Mostaganem Mostaganem Algeria; ^4^ Institute of Systems Biology (INBIOSIS) Universiti Kebangsaan Malaysia Bangi Malaysia; ^5^ College of Medicine and Health Sciences Wolaita Sodo University Wolaita Sodo Ethiopia

**Keywords:** adiposity, gut microbiota, high‐protein diet, *Lactobacillus acidophilus*, obesity, whey protein

## Abstract

This study evaluated whether *Lactobacillus acidophilus* supplementation provides additional benefits to a whey protein‐based high‐protein diet (HPD) in diet‐induced obese rats. After obesity induction, 48 male Wistar rats were assigned to four 12‐week interventions (*n* = 12/group): ND1 (140 g/kg whey protein isolate), ND2 (ND1 + *L. acidophilus* ), HPD1 (500 g/kg whey protein isolate) and HPD2 (HPD1 + *L. acidophilus* ). Supplemented groups received 5 × 10^8^ CFU/rat/day by oral gavage. Food intake, body‐weight change, regional fat mass, serum biochemical markers and gut microbiota composition were evaluated. HPD‐fed rats showed lower food intake, sustained body‐weight loss and reduced visceral and epididymal fat masses compared with rats receiving normal‐protein diets. Body‐weight trajectories differed between dietary regimens (*p* = 0.013), whereas weight loss did not differ significantly between HPD1 and HPD2 (*p* = 0.06). Fasting glycemia remained unchanged. At month 3, ND1 showed the highest triglyceride and total cholesterol concentrations, while ND2, HPD1 and HPD2 displayed lower terminal values. High‐protein feeding was associated with higher terminal urea and uric acid concentrations, whereas creatinine did not progressively increase. In the microbiota subset, HPD2 was characterized by higher relative abundances of *Lactobacillus* and *Turicibacter*. The whey protein‐based HPD was the principal factor associated with body‐weight reduction, lower adiposity and favorable lipid responses in obese rats. Under high‐protein conditions, *L. acidophilus* did not confer an additional benefit for body weight or serum lipids, although its effect on gut microbiota composition warrants further functional investigation.

## Graphical abstract

A whey protein isolate–based high‐protein diet improved metabolic health in diet‐induced obese rats by reducing body weight, adiposity, and serum lipid levels. Although *Lactobacillus acidophilus* supplementation altered gut microbiota composition by increasing beneficial bacterial taxa, it did not confer additional improvements in body weight or lipid metabolism beyond those induced by the high‐protein diet.

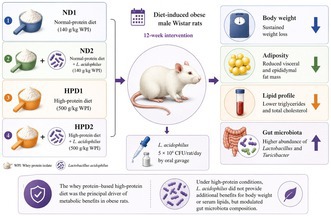

## 1 Introduction

Obesity is a chronic multifactorial metabolic disorder characterized by excessive adipose tissue accumulation and increased susceptibility to dyslipidaemia, insulin resistance, type 2 diabetes, non‐alcoholic fatty liver disease and cardiovascular complications. Its development reflects complex interactions among dietary imbalance, genetic susceptibility, altered inflammatory signaling and disruption of host–microbiota homeostasis (Blüher 2019; Qadeer et al. 2025). Because obesity is strongly influenced by modifiable dietary factors, nutritional strategies capable of reducing adiposity while improving associated metabolic disturbances remain of major experimental and clinical interest. High‐protein diets (HPDs) have been widely investigated as a nutritional strategy for obesity management because they can enhance satiety, increase diet‐induced thermogenesis, support lean tissue preservation and favorably influence lipid metabolism (Zhu et al. 2025; Khawar et al. 2025). Nevertheless, their metabolic effects depend on protein quantity and quality, the composition of the background diet and the duration of exposure. Whey protein is particularly relevant because of its high biological value, rapid digestibility and abundance of essential and branched‐chain amino acids. However, the consequences of a whey protein‐based HPD extend beyond energy balance and body‐weight regulation: increased protein intake may also modify nitrogen metabolism and alter the substrates available for intestinal microbial fermentation (Blachier et al. 2019; Yi et al. 2025). Consequently, evaluation of an HPD in obesity should integrate changes in body weight and adiposity with circulating metabolic markers and gut microbiota responses. The gut microbiota is a diet‐responsive ecosystem involved in nutrient metabolism, short‐chain fatty‐acid production, bile‐acid transformation, intestinal barrier function and regulation of low‐grade inflammation. Obesity‐associated dysbiosis has been linked to impaired metabolic homeostasis, although changes in individual taxa or phylum‐level ratios must be interpreted cautiously and in relation to host metabolic outcomes. Dietary protein can influence microbial composition and metabolic activity according to its source, digestibility and interaction with other nutrients (Blachier et al. 2019; Wu et al. 2022; Du et al. 2025). In obese individuals, a high‐protein calorie‐restricted diet has been shown to alter the gut microbiome differently from a normal‐protein dietary intervention, supporting the relevance of assessing microbial changes during protein‐based weight‐management strategies (Solfaine et al. 2024). Moreover, Li et al. (2017) reported that oat phenolic compounds combined with β‐glucan reduced hyperlipidaemia in high‐fat‐diet‐fed mice while modifying gut microbiota composition. Although this intervention differs from a whey protein‐based HPD, it supports the broader concept that diet‐induced improvements in lipid metabolism may accompany measurable remodeling of the intestinal microbiota. Probiotic supplementation may provide an additional means of modulating the metabolic and microbial consequences of dietary intervention. Among the probiotic species investigated in obesity‐related models, *Lactobacillus acidophilus* has attracted attention because of its potential to influence lipid metabolism, intestinal microbial ecology and inflammatory homeostasis. In high‐fat‐diet‐induced obese mice, *L. acidophilus* CICC 6075 attenuated body‐weight gain and fat accumulation while modifying gut microbiota composition and predicted metabolic functions (Zhang et al. 2024). Nevertheless, probiotic effects are strain‐specific and dependent on the nutritional environment in which the microorganism is administered. In particular, it remains unclear whether *L. acidophilus* provides a substantial additional metabolic benefit when combined with an already active dietary intervention such as a whey protein‐based HPD, or whether its main contribution is reflected in more limited modulation of gut microbial composition. Accordingly, the present study aimed to evaluate the effects of a whey protein‐based HPD, administered with or without *L. acidophilus* , on food intake, body‐weight evolution, visceral and epididymal adiposity, serum metabolic and nitrogen‐related markers, and gut microbiota composition in diet‐induced obese Wistar rats during a 12‐week intervention period. We hypothesized that the whey protein‐based HPD would be the principal determinant of body‐weight reduction, decreased adiposity and improved lipid profile, whereas *L. acidophilus* supplementation would exert a more limited complementary effect, primarily detectable through modulation of gut microbiota composition rather than through substantial additional weight loss.

## 2 Materials and Methods

### 2.1 Animals and Experimental Design

Forty‐eight healthy adult male Wistar rats were obtained from the Pasteur Institute, Algiers, Algeria. After quarantine and acclimatization, animals were housed in standard cages in a well‐ventilated room under controlled conditions, with a 12 h light/12 h dark cycle and free access to food and water. All procedures were performed in accordance with the National Institutes of Health guidelines for the care and use of laboratory animals and were approved by the Local Ethical Committee for Animal Care of Abdelhamid Ibn Badis University, Mostaganem, Algeria (Approval No. 2019‐013). The experiment was reported in accordance with the ARRIVE guidelines for in vivo animal research.

Obesity was induced by feeding rats a modified AIN‐93M‐based high‐fat diet (HFD) for 12 weeks. Butter was used as the specified source of animal fat at 300 g/kg diet, together with 40 g/kg soybean oil, providing an energy density of 5.31 kcal/g. At the end of the induction period, rats reaching a body weight of 350–450 g were included in the intervention phase and allocated into four groups using body‐weight‐stratified randomization (*n* = 12 per group): ND1, receiving a normal‐protein diet containing 140 g/kg whey protein isolate; ND2, receiving the same diet supplemented with *Lactobacillus acidophilus* ; HPD1, receiving a high‐protein diet containing 500 g/kg whey protein isolate; and HPD2, receiving the same high‐protein diet supplemented with *L. acidophilus* .

The four intervention diets (ND1, ND2, HPD1 and HPD2) were isocaloric, each providing 3.61 kcal/g, whereas the HFD used exclusively for obesity induction was hypercaloric. In the high‐protein diets, whey protein isolate was increased from 140 to 500 g/kg, while starch was reduced from 572.7 to 212.7 g/kg. The complete composition and energy density of all diets are presented in Table 1. In the probiotic‐supplemented groups, *L. acidophilus* was administered daily by oral gavage at a dose of 5 × 10^8^ CFU in 1 mL suspension per rat; strain origin, preparation and viable‐dose verification are described in Section 2.2.

**TABLE 1:** Composition of different dietary patterns.

Ingredients (g/kg)	HFD	ND1	ND2	HPD1	HPD2
Proteins (whey protein isolate 97%)^1^	140	140	140	500	500
Starch flour^2^	322.7	572.7	572.7	212.7	212.7
Sucrose^3^	100	100	100	100	100
Cellulose (son de blé)^4^	50	100	100	100	100
Butter (animal fat)	300	0	0	0	0
Soybean oïl^5^	40	40	40	40	40
Minerals mix, AIN 93‐M^6^	35	35	35	35	35
Vitamins mix, AIN 93‐V^7^	10	10	10	10	10
Choline^8^	2.3	2.3	2.3	2.3	2.3
Energies (kcal/g)	5310.8	3610.8	3610.8	3610.8	3610.8

*Note:* ^1^Pure Whey Isolate 97 was purchased from BULK, a company registered in England and Wales (Company No. 05654661), with a registered address at Unit 1 Gunfleet Business Park, Brunel Way, Colchester, Essex, CO4 9QX, UK, and VAT No. GB 254 5648 84. ^2–4^Reagents were obtained from Sigma‐Aldrich (S9765‐500G, S9378‐1KG, C6288‐250G, Germany). Biomérieux, Germany. ^5^Refined soybean oil was purchased from a local grocery store in Chlef, Algeria. ^6–7^MP Biomedicals, USA. BULK, a company registered in England and Wales (Company No. 05654661), with a registered address at Unit 1 Gunfleet Business Park, Brunel Way, Colchester, Essex, CO4 9QX, UK, and VAT No. GB 254 5648 84. ^8^Choline chloride (≥ 99%; catalogue No. C7017; CAS No. 67‐48‐1) was purchased from Sigma‐Aldrich, Germany.

### 2.2 Preparation of the Lactic Acid Bacterial Cell

The probiotic strain used in this experiment was isolated from Hammoum, a traditional fermented barley product collected in the Mostaganem region of Algeria, and supplied by the Laboratory of Applied Animal Physiology, Abdelhamid Ibn Badis University. The isolate was identified at species level as *Lactobacillus acidophilus* by 16S rRNA gene sequencing, followed by sequence comparison with reference sequences in the NCBI BLAST database.

For daily administration, *L. acidophilus* was inoculated into de Man, Rogosa and Sharpe (MRS) broth and incubated for 48 h at 37°C under anaerobic conditions, according to the procedure described for *L. acidophilus* administration in an experimental obesity model (Zhang et al. 2024). Bacterial cells were harvested by centrifugation at 3500 *g* for 10 min and resuspended in sterile phosphate‐buffered saline (PBS). Cell concentration was initially estimated by optical density at 600 nm, and the viable concentration of the final gavage suspension was confirmed by serial dilution and plate counting on MRS agar before administration. The suspension was then adjusted to 5 × 10^8^ CFU/mL.

Rats in the ND2 and HPD2 groups received 1 mL of freshly prepared bacterial suspension by oral gavage each day, corresponding to 5 × 10^8^ viable CFU/rat/day, throughout the 12‐week intervention period. Suspensions were prepared under sterile conditions and administered immediately after viable‐count verification to minimize losses in bacterial viability.

### 2.3 Measurement and Monitoring of Rats' Food Intake

Food intake was monitored throughout the 12‐week intervention period. Fresh powdered diets were provided three times weekly, and the amount offered and the amount remaining after 24 h were weighed using an analytical balance. Feed spillage was collected and weighed separately to improve intake accuracy. Because all experimental diets were supplied in powdered form, differences in physical texture were unlikely to affect intake measurements. Daily food intake was calculated as follows: food offered − food remaining − food spillage. When rats were individually housed, intake was expressed as g/rat/day; when rats were housed collectively, intake was calculated per cage and expressed according to the cage‐level experimental unit.

### 2.4 Body Weight Gain

Body weight was measured once weekly to minimize handling‐related stress. Body weight gain was calculated for each animal as the difference between body weight recorded on each assessment day and the baseline body weight at day 0 (measurement day − day 0) (Zhou et al. 2011).

### 2.5 Blood Collection and Biochemical Analysis

Blood samples were collected once monthly during the 12‐week intervention period, corresponding to four sampling points: baseline (M0) and months 1, 2, and 3 (M1–M3). At each sampling point, 1 mL of blood was collected from the subclavian vein of each rat according to the procedure described by Waynforth and Dunbar (1995). The sampling frequency was limited to monthly collection in order to reduce repeated handling and blood‐withdrawal‐associated stress during longitudinal biochemical monitoring. Serum was separated by centrifugation at 2500 rpm for 15 min at 4°C, aliquoted, and stored at −20°C until analysis. Serum glucose, total cholesterol, triglycerides, creatinine, uric acid, and urea concentrations were determined using a VITROS 350 automated biochemical analyzer (Ortho Clinical Diagnostics, USA). The same sampling schedule and analytical procedures were applied to all experimental groups to minimize differential procedural effects among treatments.

### 2.6 Body Fat Measurement

At the termination of the 12‐week dietary intervention period, visceral and epididymal adipose tissue depots were carefully dissected from each rat under standardized conditions. After removal of adherent non‐adipose tissues, each fat depot was immediately weighed separately using an analytical balance, and the results were expressed as wet tissue mass in grams (g) per rat. Visceral and epididymal fat masses were analyzed as indicators of regional adipose tissue accumulation, with each individual rat considered as the experimental unit for statistical comparisons among dietary groups.

### 2.7 DNA Extraction and PCR Amplification

The contents of the large intestine from three rats per group were collected into 2 mL cryotubes containing PBS, kept briefly at room temperature, followed by storage at −80°C until DNA was extracted. Electrophoresis on a 1% agarose gel was used to assess the concentration and purity of DNA, and samples were then diluted to 1 ng/mL using sterile water. Total microbial DNA was extracted from intestinal contents using a commercial mini stool DNA kit (Qiagen, Hilden, Germany) as directed by the manufacturer. The V3–V4 region of the bacterial 16S rRNA gene was amplified with universal barcoded primers 338F (5′‐ACTCCTACGGGAGGCAGCAG‐3′) and 806R (5′‐GGACTACHVGGGTWTCTAAT‐3′). PCR reactions (20 μL) contained 1 FastPf buffer, 250 μM dNTP, 0.1 μM of each primer, 1 U FastPfu DNA polymerase (Beijing TransGen Biotech, China) and 10 ng template DNA, under the following cycling conditions: 95°C for 2 min; 30 cycles at 95°C for 30 s, 55°C for 30 s and 72°C for 30 s; and a final extension at 72°C for 5 min. Amplicons were spotted on 2% agarose gels, purified, and grouped together at equal concentrations to create a library. Libraries were end‐repaired, A‐tailed, ligated to Illumina adapters, quantified by Qubit and real‐time PCR, and sequenced on an Illumina paired‐end platform (2 × 250 bp).

### 2.8 Sequencing Data Processing

Samples were demultiplexed based on their unique barcodes, followed by the removal of both barcode and primer sequences from the paired‐end reads. Assembly of these paired‐end reads was carried out using FLASH (v1.2.7), yielding spliced sequences termed raw tags. To isolate high‐quality clean tags, the raw tags underwent quality‐control filtering via the QIIME (v1.7.0) pipeline. Downstream sequence processing of all effective tags was executed using UPARSE (v7.0.1090). Operational taxonomic units (OTUs) were generated by clustering sequences with a minimum similarity threshold of 97%, after which representative sequences were chosen for taxonomic classification. Annotation across taxonomic ranks was performed through the Mothur method within QIIME (v1.7.0) utilizing the SILVA SSUrRNA database, applying a confidence interval of 0.8 to 1. To determine phylogenetic relationships, the representative OTU sequences were aligned using MUSCLE (v3.8.31). Finally, alpha and beta diversity metrics were computed based on an OTU abundance dataset normalized to the minimum sequencing depth across samples.

### 2.9 Statistical Analyses

Statistical analyses were performed using GraphPad Prism version 9.0. Two‐way analysis of variance with repeated measures (two‐way RM ANOVA) was applied for mean comparisons, with statistical significance set at *p* < 0.05, followed by Tukey's post hoc test for multiple comparisons or correlation analyses, as appropriate. GraphPad Prism was also used to generate curves and histograms.

## 3 Results

### 3.1 Composition and Consumption of Food

**FIGURE 1:**
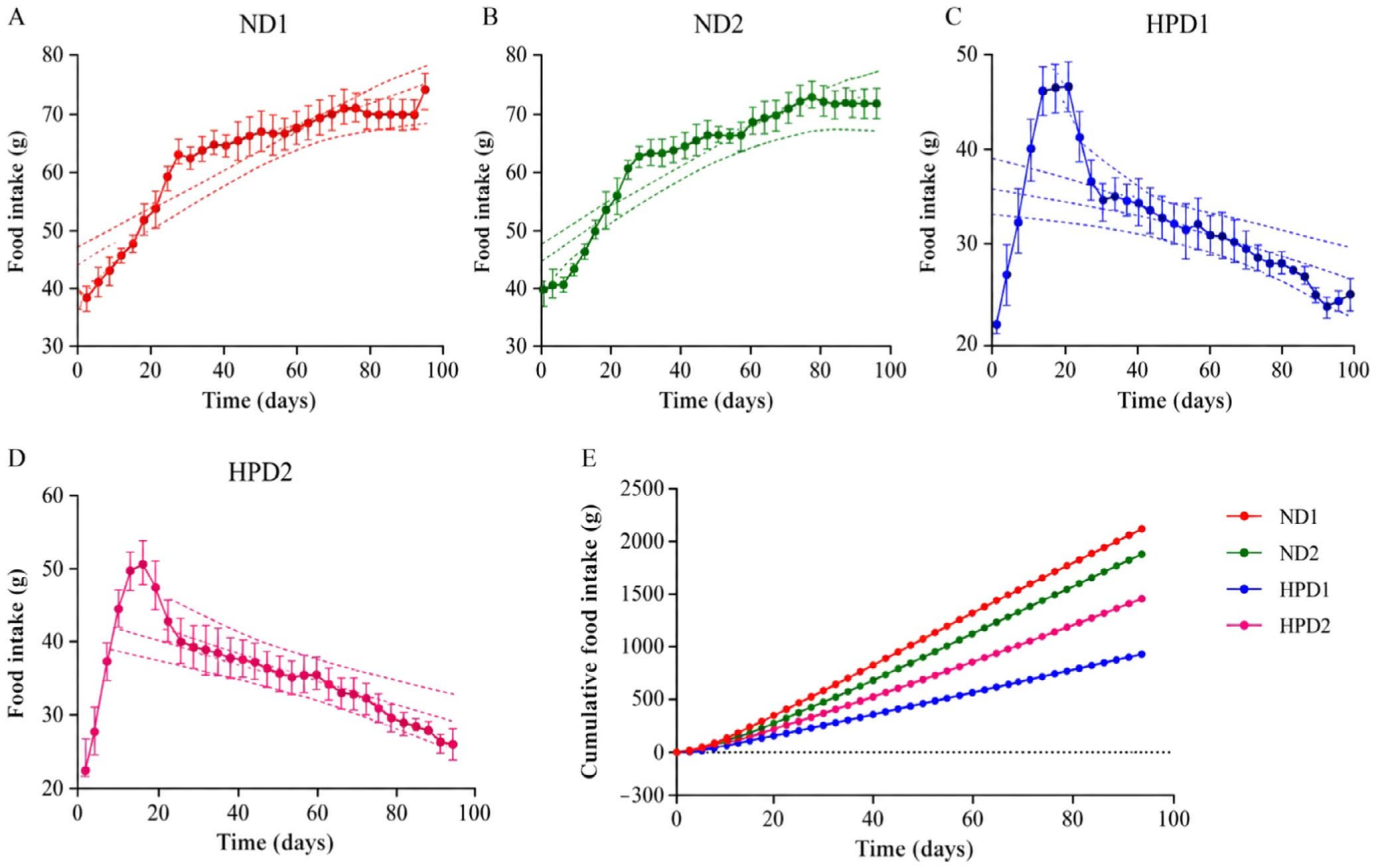
The effects of diet composition on food intake. (A) ND1: 14% whey protein, (B) ND2: 14% whey protein + *L. acidophilus* , (C) HPD1: 50% whey protein, (D) HPD2: 50% whey protein + *L. acidophilus* , (E) cumulative consumption over 90 days of treatment, where data points represent the mean ± SD.

The composition and energy density of the obesity‐induction and intervention diets are presented in Table 1. During the 12‐week intervention period, food intake displayed distinct trajectories according to dietary protein content (Figure 1). Rats receiving the normal‐protein diets (ND1 and ND2) maintained higher daily food intake throughout the experiment, whereas rats receiving the high‐protein diets (HPD1 and HPD2) exhibited an initial increase followed by a sustained decline in intake until day 90.

This pattern was reflected in cumulative food intake, which was lower in both high‐protein groups than in the normal‐protein groups at the end of the intervention period (Figure 1E). Within each protein level, the trajectories were broadly similar: ND2 showed a modestly lower cumulative intake than ND1, whereas HPD2 showed only a slight numerical increase compared with HPD1. Thus, the reduction in food consumption was principally associated with the whey protein‐based high‐protein diet, while *Lactobacillus acidophilus* supplementation did not produce a prominent additional effect on this outcome.

### 3.2 Body Weight Loss

**FIGURE 2:**
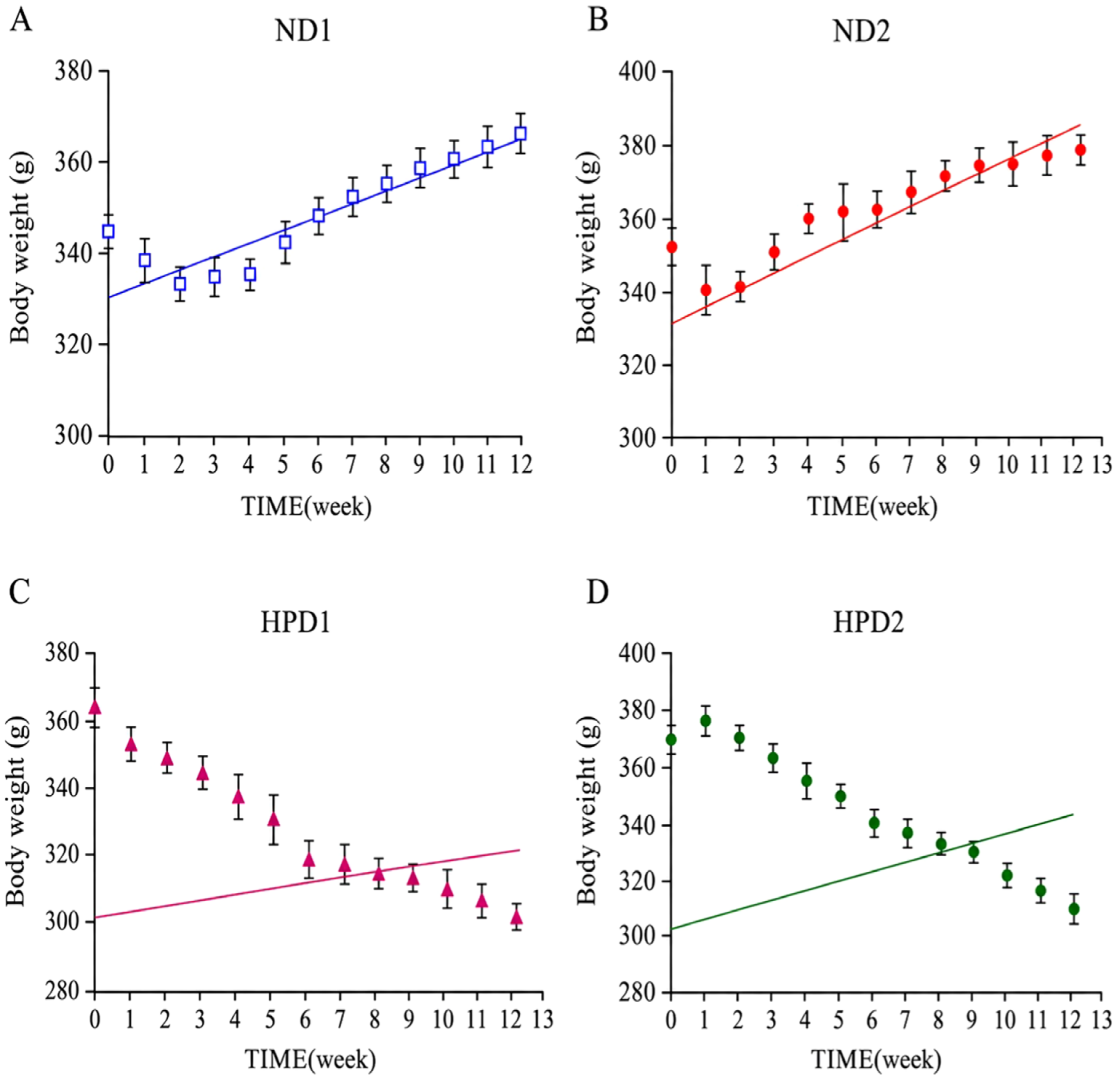
Correlation of body weight gain (g) of rats between four groups: (A) ND1: 14% whey protein, (B) ND2: 14% whey protein + *L. acidophilus* , (C) HPD1: 50% whey protein, (D) HPD2: 50% whey protein + *L. acidophilus* , where data points represent the mean ± SD and *p* = 0.013.

Figure 2 illustrates body‐weight evolution in rats subjected to different dietary interventions over the 12‐week experimental period. Distinct patterns of body‐weight change were observed between rats receiving normal diets (ND1 and ND2) and those receiving high‐protein diets (HPD1 and HPD2). Rats in the ND1 and ND2 groups exhibited a gradual and significant increase in body weight throughout the experiment (Figure 2A,B). Body‐weight trajectories were similar between ND1 and ND2, with no statistically significant differences between these groups. A strong positive correlation was observed between food intake and body‐weight gain in the normal‐diet groups (*p* < 0.0001). In contrast, rats fed high‐protein diets (HPD1 and HPD2) showed a progressive reduction in body weight beginning in the third week of intervention (Figure 2C,D). Weight loss was more pronounced in the HPD1 group than in HPD2, although this difference did not reach statistical significance (*p* = 0.06). No significant correlation was observed between food intake and body‐weight gain in HPD1 (*p* = 0.18), indicating a dissociation between intake and body‐weight evolution under high‐protein feeding. Comparison between dietary regimens revealed a statistically significant difference in body‐weight trajectories between the ND and HPD groups (*p* = 0.013), confirming a distinct effect of dietary protein content on body‐weight regulation.

### 3.3 Effect of Food Consumption on Blood Lipids and Glycemia

The effects of dietary interventions on glycemia and blood lipid parameters were evaluated at monthly intervals over the 12‐week intervention period across the four experimental groups (Table 2). Fasting glycemia remained stable in all groups throughout the study, with no statistically significant within‐group changes over time (ND1: *p* = 0.965; ND2: *p* = 0.141; HPD1: *p* = 0.501; HPD2: *p* = 0.126). Between‐group comparisons also revealed no significant differences in glycemic levels at any time point. These results indicate that neither dietary protein level nor *Lactobacillus acidophilus* supplementation significantly affected fasting glycemia under the present experimental conditions.

**TABLE 2:** Comparison of means, standard errors, and *p*‐values of biochemical blood parameters among groups over the 3‐month treatment period.

Time (months)	M0	M1	M2	M3	*p* ^d^
**Glycemia (mg/dL)**					
ND1	117.50 ± 1.27	111.25 ± 0.14	113.00 ± 2.60	115.75 ± 1.27	0.965^c^
ND2	101.00 ± 2.23	114.00 ± 0.93	114.75 ± 2.82	120.25 ± 2.05	0.141^c^
HPD1	98.25 ± 1.36	101.25 ± 2.75	100.75 ± 2.98	99.75 ± 1.40	0.501^c^
HPD2	107.50 ± 1.59	117.25 ± 2.21	103.25 ± 2.75	104.75 ± 1.40	0.126^c^
*p* ^e^	0.14^c^	0.96^c^	0.25^c^	0.09^c^	
**Triglycerides (g/L)**					
ND1	0.78^a^ ± 0.07	0.52^b^ ± 0.09	1.44^a^ ± 0.17	1.51^a^ ± 0.18	< 0.0001
ND2	0.82^a^ ± 0.04	0.73^a^ ± 0.03	0.43^b^ ± 0.06	0.35^a^ ± 0.01	< 0.0001
HPD1	0.79^a^ ± 0.07	0.76^a^ ± 0.22	0.40^b^ ± 0.03	0.36^a^ ± 0.01	0.0002
HPD2	0.76^a^ ± 0.04	0.71^a^ ± 0.07	0.40^b^ ± 0.03	0.37^a^ ± 0.02	< 0.0001
*p* ^e^	0.62^c^	0.08^c^	0.001	< 0.0001	
**Cholesterol (g/L)**					
ND1	0.96^a^ ± 0.06	0.67^b^ ± 0.05	0.92^a^ ± 0.29	1.31^b^ ± 0.14	0.001
ND2	0.90^a^ ± 0.08	0.72^a^ ± 0.12	0.61^b^ ± 0.03	0.53^b^ ± 0.05	< 0.0001
HPD1	0.93^a^ ± 0.07	0.61^b^ ± 0.05	0.85^a^ ± 0.05	0.65^a^ ± 0.13	0.0001
HPD2	0.95^a^ ± 0.06	0.66^b^ ± 0.07	0.84^a^ ± 0.08	0.69^b^ ± 0.03	0.0001
*p* ^e^	0.36^c^	0.07^c^	< 0.0001	< 0.0001	
**Creatinine (mg/L)**					
ND1	6.69^a^ ± 0.57	3.74^b^ ± 0.13	3.81^b^ ± 0.15	4.28^c^ ± 0.11	< 0.0001
ND2	6.63^a^ ± 0.52	3.61^b^ ± 0.12	4.93^a^ ± 0.58	4.60^a^ ± 0.21	< 0.0001
HPD1	6.72^a^ ± 0.51	3.09^b^ ± 0.23	4.57^c^ ± 0.29	4.04^a^ ± 0.28	< 0.0001
HPD2	6.52^a^ ± 0.67	3.99^b^ ± 0.21	5.53^a^ ± 0.69	4.34^c^ ± 0.05	< 0.0001
*p* ^e^	0.96^c^	0.0001	0.002	0.009	
**Uric acid (mg/L)**					
ND1	0.26 ± 0.05	0.31 ± 0.01	0.35 ± 0.15	0.40 ± 0.1	0.08^c^
ND2	0.24 ± 0.04	0.29 ± 0.03	0.33 ± 0.05	0.38 ± 0.01	0.311^c^
HPD1	0.37^a^ ± 0.06	0.42^a^ ± 0.12	0.46^a^ ± 0.03	0.51^a^ ± 0.03	0.002
HPD2	0.41^a^ ± 0.03	0.46^a^ ± 0.07	0.50^a^ ± 0.03	0.55^a^ ± 0.02	0.0005
*p* ^e^	0.91^c^	0.009	0.03	0.01	
**Urea (g/L)**					
ND1	0.32^a^ ± 0.03	0.30^a^ ± 0.01	0.35^a^ ± 0.11	0.37^b^ ± 0.12	< 0.0001
ND2	0.33^a^ ± 0.06	0.33^b^ ± 0.01	0.31^b^ ± 0.09	0.29^a^ ± 0.09	0.003
HPD1	0.32^a^ ± 0.04	0.39^a^ ± 0.03	0.48^a^ ± 0.10	0.58^b^ ± 0.07	< 0.0001
HPD2	0.33^a^ ± 0.03	0.47^a^ ± 0.02	0.52^a^ ± 0.07	0.62^b^ ± 0.10	< 0.0001
*p* ^e^	0.13^c^	0.09^c^	0.02	0.04	

*Note:* ND1: 14% whey protein, B; ND2: 14% whey protein + *L. acidophilus* , C; HPD1: 50% whey protein, D; HPD2: 50% whey protein + *L. acidophilus* . M0: is the baseline (starting point), M1: the first month, M2: the second month, M3: the third month.

^a^ Highly significant.

^b^ Significant.

^c^ Not significant.

^d^ The intra‐group comparison over the 12‐week treatment period.

^e^ The inter‐group comparisons across treatment groups on a monthly basis.

In contrast, triglyceride concentrations exhibited marked group‐dependent variations. The ND1 group showed a significant increase in triglyceride levels over time, reaching 1.51 ± 0.18 g/L at M3 compared with 0.78 ± 0.07 g/L at baseline (*p* < 0.0001). Conversely, the ND2, HPD1 and HPD2 groups displayed significant decreases from 0.82 ± 0.04, 0.79 ± 0.07 and 0.76 ± 0.04 g/L at M0 to 0.35 ± 0.01, 0.36 ± 0.01 and 0.37 ± 0.02 g/L at M3, respectively (ND2: *p* < 0.0001; HPD1: *p* = 0.0002; HPD2: *p* < 0.0001). Between‐group differences were not significant at M0 or M1, but became significant at M2 (*p* = 0.001) and M3 (*p* < 0.0001). Therefore, terminal triglyceride concentrations were lower in ND2, HPD1 and HPD2 than in the unsupplemented normal‐protein group (ND1), with ND2 presenting the lowest numerical value at M3.

Total cholesterol levels also differed significantly among groups. Cholesterol concentrations increased significantly in the ND1 group, from 0.96 ± 0.06 g/L at M0 to 1.31 ± 0.14 g/L at M3 (*p* = 0.001), whereas significant decreases were observed in ND2, HPD1 and HPD2, reaching terminal values of 0.53 ± 0.05, 0.65 ± 0.13 and 0.69 ± 0.03 g/L, respectively (ND2: *p* < 0.0001; HPD1: *p* = 0.0001; HPD2: *p* = 0.0001). Between‐group differences became significant at M2 and remained significant at M3 (*p* < 0.0001 for both sampling points). As observed for triglycerides, ND2 showed the lowest numerical cholesterol concentration at M3, followed by HPD1 and HPD2.

### 3.4 Effect of Food Consumption on Uric Acid, Urea, and Creatinine

The effects of dietary interventions on serum creatinine, uric acid and urea concentrations were evaluated monthly throughout the 12‐week intervention period (Table 2). Serum creatinine concentrations varied significantly over time in all experimental groups (ND1, ND2, HPD1 and HPD2: *p* < 0.0001). In all groups, creatinine values decreased from baseline at M1 and subsequently increased moderately at M2 and M3, while remaining below their respective baseline values at the end of the experiment. At M3, creatinine concentrations were 4.28 ± 0.11 mg/L in ND1, 4.60 ± 0.21 mg/L in ND2, 4.04 ± 0.28 mg/L in HPD1 and 4.34 ± 0.05 mg/L in HPD2. Between‐group differences were not significant at baseline (*p* = 0.96), but became significant at M1 (*p* = 0.0001), M2 (*p* = 0.002) and M3 (*p* = 0.009). Uric acid concentrations remained statistically unchanged over time in the normal‐protein groups (ND1: from 0.26 ± 0.05 to 0.40 ± 0.10 mg/L, *p* = 0.08; ND2: from 0.24 ± 0.04 to 0.38 ± 0.01 mg/L, *p* = 0.311). In contrast, significant increases were observed in both high‐protein groups, with uric acid increasing from 0.37 ± 0.06 to 0.51 ± 0.03 mg/L in HPD1 (*p* = 0.002) and from 0.41 ± 0.03 to 0.55 ± 0.02 mg/L in HPD2 (*p* = 0.0005). No significant difference was observed among groups at baseline (*p* = 0.91), whereas between‐group differences were detected at M1 (*p* = 0.009), M2 (*p* = 0.03) and M3 (*p* = 0.01). Numerically, HPD2 exhibited the highest terminal uric acid concentration, followed by HPD1.

Serum urea concentrations also differed according to dietary intervention. Urea increased over time in ND1, from 0.32 ± 0.03 g/L at M0 to 0.37 ± 0.12 g/L at M3 (*p* < 0.0001), whereas ND2 showed a reduction from 0.33 ± 0.06 to 0.29 ± 0.09 g/L (*p* = 0.003). The greatest terminal urea concentrations were observed in the high‐protein groups, increasing from 0.32 ± 0.04 to 0.58 ± 0.07 g/L in HPD1 and from 0.33 ± 0.03 to 0.62 ± 0.10 g/L in HPD2 (*p* < 0.0001 for both groups). Between‐group differences were not significant at M0 or M1, but became significant at M2 (*p* = 0.02) and M3 (*p* = 0.04).

### 3.5 Effect of Food Consumption on the Visceral and Epididymis Fat Weight

**FIGURE 3:**
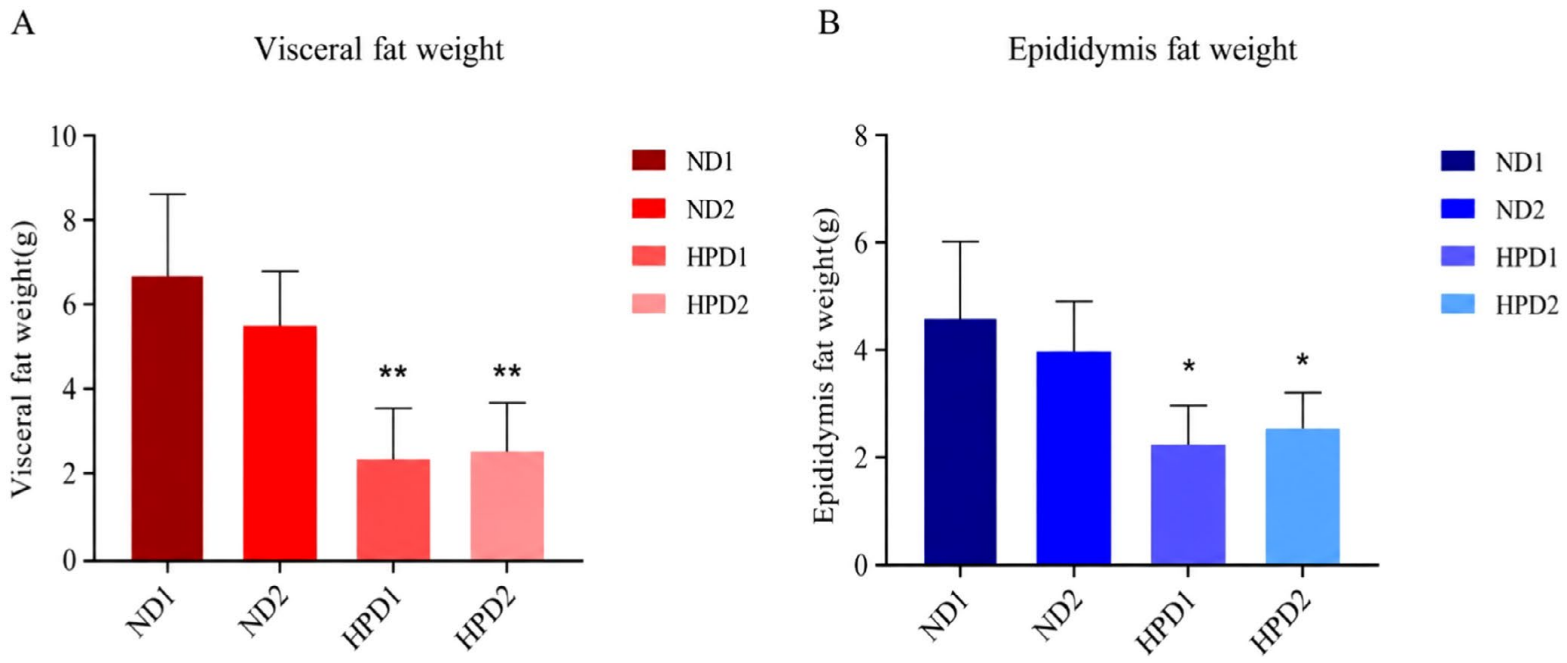
Visceral fat weight (A) and epididymis fat weight (B): Normal diet (ND1); normal diet + *Lactobacillus acidophilus* (ND2); high protein diet (HPD1); high protein diet + *Lactobacillus acidophilus* (HPD2) (*p < 0.05 significant; **p < 0.01 highly significant).

The effects of dietary interventions on visceral and epididymal adipose tissue accumulation were evaluated in rats fed a normal‐protein diet or a high‐protein diet, with or without *Lactobacillus acidophilus* supplementation (Figure 3). Visceral fat mass differed significantly among the experimental groups (*p* = 0.0012). Rats in the ND1 and ND2 groups exhibited higher visceral fat masses (6.73 ± 0.70 g and 5.64 ± 1.00 g, respectively) than rats in the HPD1 and HPD2 groups (2.33 ± 0.99 g and 2.51 ± 0.56 g, respectively). Thus, the lowest visceral fat accumulation was observed in animals receiving the high‐protein diets. Similarly, epididymal fat mass differed significantly according to dietary intervention (*p* = 0.01). Compared with ND1 (4.57 ± 0.98 g), epididymal fat mass was significantly lower in HPD1 (2.27 ± 0.36 g; *p* = 0.014) and HPD2 (2.64 ± 0.20 g; *p* = 0.038).

### 3.6 Effect of Diets on Gut Microbiota Composition

**FIGURE 4:**
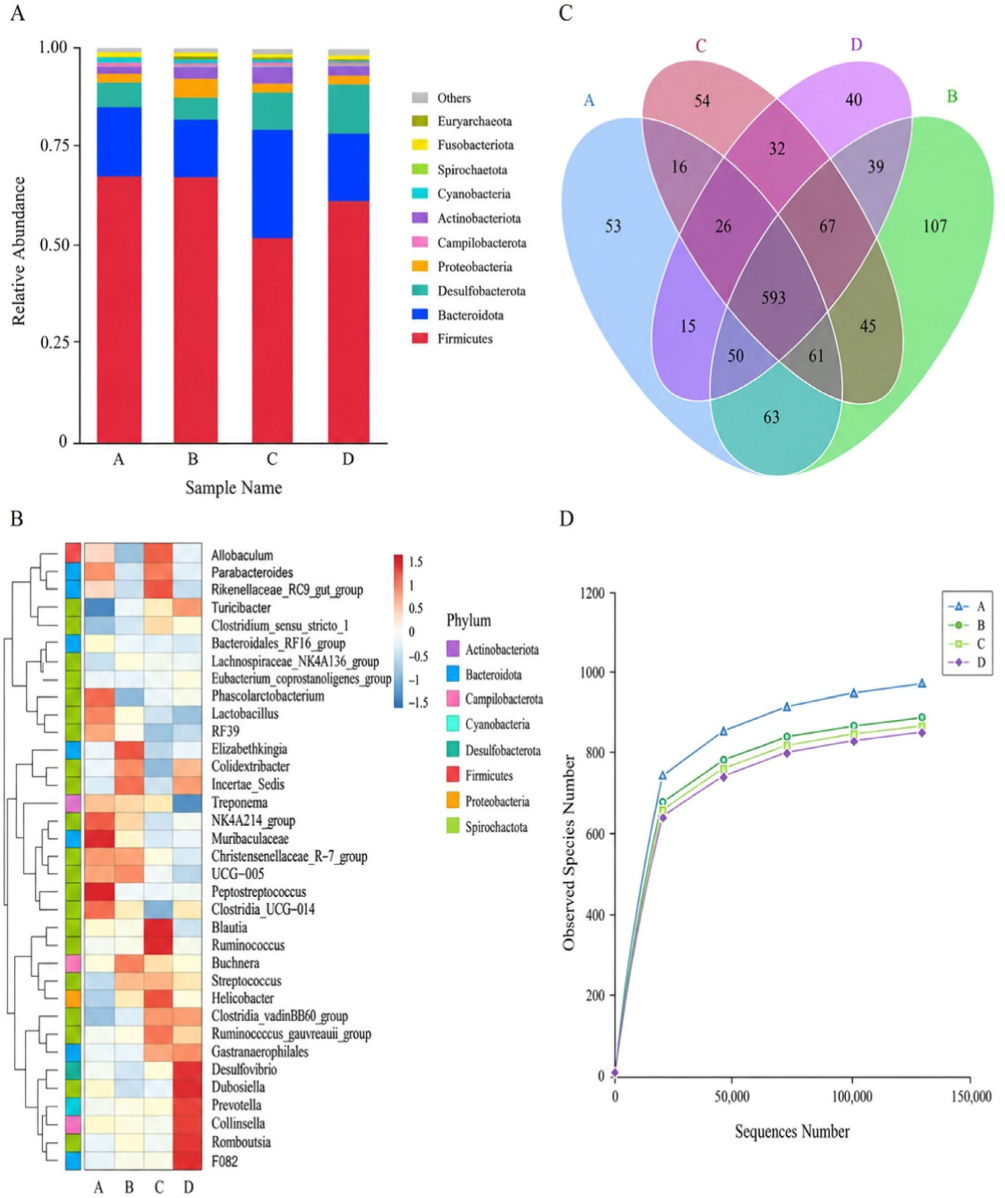
Gut microbiota analysis from feces of rat fed with a normal diet, high protein diet with and without *Lactobacillus acidophilus* (A: ND1; B: ND2; C: HPD1; D: HPD2) as determined by the relative abundance of bacterial diversity at phylum (A), taxonomic abundance of bacterial diversity cluster heatmap at phylum and genus (B), Venn of the OTUs in different treatments (C), rarefaction curves representing the biodiversity of the gut microbiota (D).

**FIGURE 5:**
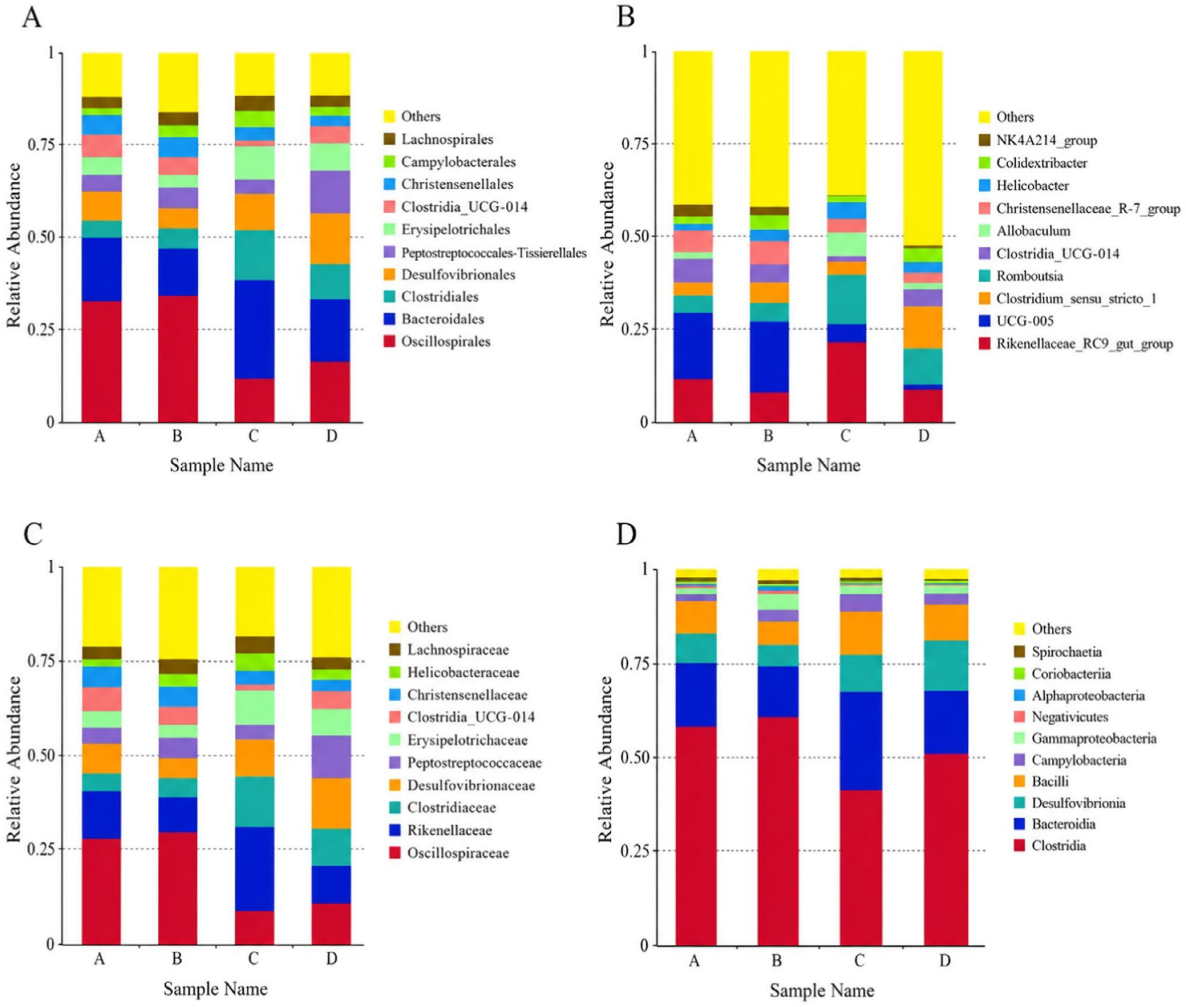
The relative abundance of the top ten families (A), genera (B), orders (C), and classes (D) in rats fed a regular or high protein diet with or without *Lactobacillus acidophilus* . The various groupings are displayed on the horizontal axis. The vertical axis, represented by columns of varying colors, shows the relative abundance of the various bacteria.

The composition of microbial communities was assessed using diversity and richness indices. Simpson and Shannon indices (Table 3) were utilized to assess gut microbiota diversity and community structure, whereas Chao1, ACE, and Observed_species captured richness, and Good's Coverage estimated sequencing depth. All sample categories showed Coverage values above 97%, indicating high sampling completeness and reliable characterization of the gut microbiota. Richness metrics (Observed species, Chao1, ACE) did not differ significantly among ND1, ND2, HPD1, and HPD2 (*p* = 0.69, 0.76, and 0.82, respectively), whereas Shannon (*p* = 0.049) and Simpson (*p* = 0.05) indices revealed modest but statistically detectable differences in alpha diversity/evenness (Figures 4A–C and 5) (Korotetskiy et al. 2025).

**TABLE 3:** Alpha diversity indices of gut microbiota diversity in rats of ND1, ND2 and HPD1, HPD2 groups.

Sample	Observed_species	Shannon	Simpson	chao1	ACE
A: ND1	877 ± 8.9	6.333 ± 0.8	0.959 ± 0.06	905.835 ± 7.56	905.905 ± 9.33
B: ND2	1025 ± 9.91	6.671 ± 0.61	0.969 ± 0.03	1082.028 ± 6.6	1094.381 ± 10.1
C: HPD1	894 ± 7.68	5.839 ± 0.55	0.952 ± 0.02	942.533 ± 8.2	943.516 ± 9.99
D: HPD2	862 ± 6.78	6.325 ± 0.73	0.964 ± 0.09	897.577 ± 8.99	897.705 ± 6.85
*p*	0.69	0.049	0.05	0.76	0.82

*Note:* Normal diet (ND1); normal diet + Lactobacillus *acidophilus* (ND2); high protein diet (HPD1); high protein diet + *Lactobacillus acidophilus* (HPD2) (*p* < 0.05; indicates a significant difference; *p* > 0.05; indicates no significant difference).

At phylum level, ND1 displayed a higher Firmicutes/Bacteroidetes ratio (~68%) than HPD groups (52%–54%; *p* < 0.01), whereas Bacteroidetes increased to ~26% in HPD1 and Proteobacteria were less abundant in HPD1–HPD2 (~2%) than in ND2 (~5%). Actinobacteria remained low but increased modestly to ~1% in HPD2 and ND2. At the class level, Clostridia were dominant in ND2 (61%), reflecting stronger Firmicutes dominance, while high‐protein diets shifted the Firmicutes community and lowered the Firmicutes/Bacteroidetes ratio, a pattern often linked to improved metabolic and inflammatory profiles. *L. acidophilus* supplementation significantly increased the relative abundance of several families, including Bacteroidaceae, Eubacteriaceae, Prevotellaceae, Clostridiaceae and Enterococcaceae (Figure 5). Clostridia and Bacilli remained the predominant Firmicutes classes in all groups, but their proportions shifted with diet: ND1 and ND2 were characterized by Clostridia 58%–61% and Bacilli 6%–9%, whereas HPD1 and HPD2 showed lower Clostridia (42%–51%) and somewhat higher Bacilli (10%–12%), with Clostridia being significantly more abundant in HPD groups than in ND groups (*p* = 0.03). At the genus level, *Allobaculum* was significantly enriched in HPD1 (6%) and HPD2 (5%) compared with ND groups (*p* = 0.02), and both *Lactobacillus* and *Turicibacter* reached higher relative abundance (~5%) in HPD2 than in the other groups (*p* < 0.01).

## 4 Discussion

### 4.1 Composition and Consumption of Food

The reduction in food intake observed in the HPD groups indicates that dietary protein level was the principal factor influencing feeding behavior in the present experiment. Rats receiving the whey protein‐based HPD exhibited lower daily and cumulative food intake than rats receiving the normal‐protein diets, a response consistent with previous evidence that protein‐rich diets, particularly those containing rapidly digestible dairy proteins, can reduce voluntary food consumption and contribute to body‐weight management (French et al. 2017; Zhou et al. 2009; Blachier et al. 2019).

In contrast, the influence of *Lactobacillus acidophilus* supplementation on food intake appeared limited. Within the same protein level, the intake trajectories of supplemented and non‐supplemented groups were broadly similar, indicating that the probiotic did not produce a prominent additional effect on food consumption. This interpretation is consistent with reports showing that the metabolic effects of Lactobacillus strains are often more evident for body‐weight regulation, lipid metabolism and gut microbiota composition than for direct modification of food intake (Ondee et al. 2021; Reamtong et al. 2021).

### 4.2 Body Weight Loss

The present findings indicate that the whey protein‐based high‐protein diet was the principal determinant of body‐weight reduction in obese rats. Whereas rats receiving the normal‐protein diets progressively gained weight during the intervention period, those receiving HPD1 or HPD2 showed sustained body‐weight loss from the third week onward. This response was accompanied by lower food intake and reduced visceral and epididymal fat masses in the HPD groups, indicating that the improvement in body‐weight status was consistent with reduced food consumption and lower regional adiposity. Similar beneficial effects of protein‐enriched diets on body‐weight regulation and adipose tissue accumulation have been reported in obesity‐related experimental models (Aparicio, Sánchez, et al. 2013; Sousa et al. 2018; Blachier et al. 2019). Importantly, the present results do not provide evidence that *Lactobacillus acidophilus* enhanced the body‐weight‐reducing effect of the HPD. Although both high‐protein groups exhibited weight loss, the reduction was numerically greater in HPD1 than in HPD2, and the difference between these groups was not statistically significant (*p* = 0.06). Therefore, the body‐weight response should be attributed primarily to the high‐protein dietary intervention, while any complementary contribution of *L. acidophilus* is more appropriately considered in relation to microbiota‐related outcomes rather than additional weight loss. Because energy expenditure, fecal energy loss, and appetite‐regulatory hormones were not measured, the mechanisms responsible for HPD‐associated body‐weight reduction cannot be directly established from the present data. Nevertheless, the concordant reduction in food intake, body weight, and regional adipose tissue mass supports the conclusion that the whey protein‐based HPD was effective in improving body‐weight outcomes in diet‐induced obese rats.

### 4.3 Effect of Food Consumption on Blood Lipids and Glycemia

Fasting glycemia remained unchanged throughout the intervention period in all experimental groups, indicating that neither the whey protein‐based high‐protein diet nor *Lactobacillus acidophilus* supplementation markedly affected basal glucose concentrations under the present conditions. This result may be explained by the fact that the animals, although obese, were not selected for established hyperglycemia or diabetes. Similar observations have been reported in non‐diabetic experimental models, in which nutritional or probiotic interventions may improve adiposity and circulating lipid parameters without producing measurable changes in fasting glycemia (Stengel et al. 2013; de Almeida et al. 2022). Because insulin concentrations, glucose tolerance and insulin‐resistance indices were not assessed in the present study, no conclusion can be drawn regarding insulin sensitivity or glucose homeostasis beyond fasting glycemia. In contrast, serum triglyceride and total cholesterol concentrations responded clearly to the dietary interventions. The unsupplemented normal‐protein group (ND1) showed the least favorable lipid profile at the end of the experiment, whereas ND2, HPD1 and HPD2 displayed lower terminal triglyceride and cholesterol concentrations. The improvement observed in the HPD groups is consistent with their lower food intake, body‐weight reduction and decreased visceral and epididymal adipose tissue masses, suggesting that the lipid response accompanied the overall improvement in adiposity induced by high‐protein feeding. Previous experimental evidence similarly indicates that protein‐enriched dietary strategies can improve circulating lipid profiles in obesity‐related models, particularly when reductions in body weight and adipose tissue accumulation occur concurrently (Aparicio, Sánchez, et al. 2013; Sousa et al. 2018; Blachier et al. 2019). The favorable lipid profile observed in ND2 also suggests that *L. acidophilus* supplementation may influence lipid metabolism under normal‐protein conditions. However, under high‐protein conditions, terminal triglyceride and cholesterol concentrations were comparable between HPD1 and HPD2, indicating that probiotic supplementation did not produce a clear additional lipid‐lowering effect beyond that associated with the HPD alone. Thus, the present findings support a predominant role of the high‐protein diet in improving adiposity‐related outcomes, while the contribution of *L. acidophilus* to circulating lipid responses appears dependent on dietary context. Since lipoprotein fractions, hepatic lipid accumulation, inflammatory mediators and microbial metabolites were not measured, the biological mechanisms underlying these lipid changes remain to be confirmed.

### 4.4 Effect of Food Consumption on Uric Acid, Urea, and Creatinine

The higher terminal urea concentrations observed in HPD1 and HPD2 are biologically consistent with the increased nitrogen load imposed by a whey protein‐based high‐protein diet. Urea is the principal circulating nitrogenous end‐product generated during dietary and endogenous protein catabolism, and its production generally increases when amino‐acid degradation is enhanced (Weiner et al. 2015; Ko et al. 2020). Thus, the elevation in serum urea in HPD‐fed rats may primarily reflect greater amino‐acid turnover and renal nitrogen excretion rather than renal injury per se. Nevertheless, the concurrent increase in uric acid in both HPD groups indicates that high‐protein feeding modified several nitrogen‐ or purine‐related biochemical markers. Because uric acid originates from purine catabolism rather than directly from amino‐acid deamination, its increase should be interpreted cautiously and cannot be attributed solely to increased protein catabolism without additional metabolic measurements. The absence of a progressive increase in serum creatinine provides no biochemical evidence of overt deterioration in renal filtration during the 12‐week intervention. However, this observation should not be interpreted as definitive evidence of renal safety. In a 12‐week experiment in Wistar rats, Aparicio, Nebot, et al. (2013) reported that a high‐protein diet adversely affected urinary and renal morphological parameters, including glomerular and mesangial changes, even when evaluation was not limited to circulating markers. More broadly, high dietary protein intake has been associated with renal adaptive responses, including increased filtration demand and potential hyperfiltration, although the clinical significance of these changes depends on exposure duration, protein source and baseline renal status (Kamper and Strandgaard 2017; Ko et al. 2020). Conversely, systematic evidence in healthy adults indicates that higher protein intake does not necessarily produce an adverse change in glomerular filtration rate over the duration of controlled interventions, highlighting the need for cautious interpretation across populations and models (Devries et al. 2018). In the present experiment, *Lactobacillus acidophilus* supplementation did not attenuate the biochemical response associated with high‐protein feeding. At the end of the intervention, HPD2 exhibited numerically higher urea and uric acid concentrations than HPD1, whereas creatinine concentrations were of similar magnitude in the two HPD groups. Therefore, the present findings do not support a nephroprotective effect of *L. acidophilus* under high‐protein conditions. Equally, because renal histopathology, urine protein excretion, albuminuria, urinary nitrogen excretion and direct measures of glomerular filtration were not assessed, no conclusion regarding renal toxicity can be established from serum urea, uric acid and creatinine alone.

### 4.5 Effect of Food Consumption on the Visceral and Epididymis Fat Weight

The marked reduction in visceral and epididymal fat mass observed in the HPD groups indicates that the whey protein‐based high‐protein diet was the principal factor associated with lower regional adipose tissue accumulation in obese rats. This finding is consistent with the concomitant reduction in food intake and body weight observed in HPD‐fed animals, suggesting that the decrease in adiposity formed part of the overall response to the protein‐enriched dietary intervention. In support of this interpretation, Song et al. (2023) reported that increasing dietary protein content in high‐fat‐fed rats significantly reduced body‐weight gain, epididymal adipose tissue mass and adipocyte size, demonstrating that high dietary protein can limit regional fat accumulation in an experimental obesity model. The relevance of whey protein in this context is supported by evidence indicating that whey‐based interventions may contribute to reductions in body fat and improvement of body composition; however, the magnitude of these effects depends on experimental conditions, background diet and metabolic status (Giglio et al. 2023; Zhao et al. 2025). In the present study, both HPD1 and HPD2 showed markedly lower visceral and epididymal fat masses than the normal‐protein groups, supporting the interpretation that increased dietary whey protein content was closely associated with reduced adipose tissue accumulation after obesity induction. Although some experimental studies have shown that selected *Lactobacillus acidophilus* strains can attenuate fat accumulation in high‐fat‐diet‐induced obesity models, such effects are strain‐ and context‐dependent. For example, Zhang et al. (2024) reported reduced fat accumulation in HFD‐fed mice receiving *L. acidophilus* CICC 6075. In the present experiment, however, visceral and epididymal fat masses did not differ significantly between HPD1 and HPD2. Therefore, the current results do not demonstrate an additional anti‐adiposity effect of *L. acidophilus* when administered with the whey protein‐based HPD.

### 4.6 Effect of Diets on the Intestinal Microbiota Composition

The present results show that the dietary interventions modified gut microbiota composition more clearly than overall microbial richness. Although Observed species, Chao1 and ACE indices did not differ significantly among groups, Shannon and Simpson indices revealed modest differences in community diversity and evenness. This pattern suggests that the interventions selectively remodeled the distribution of intestinal bacterial taxa rather than producing a major gain or loss of species richness. Such an effect is biologically plausible, because both the quantity and source of dietary protein can alter the substrates reaching the colon, thereby influencing microbial composition and fermentation activity (Bartlett and Kleiner 2022; Wu et al. 2022). At the phylum level, rats receiving the high‐protein diets showed a lower Firmicutes/Bacteroidetes ratio than rats in the ND1 group. Although a higher Firmicutes/Bacteroidetes ratio has frequently been associated with obesity‐related dysbiosis, its use as a standalone biomarker is controversial because findings vary according to diet, host characteristics, sequencing procedures and analytical methods (Magne et al. 2020; Karačić et al. 2024; Meng et al. 2024). Therefore, the lower ratio observed in HPD‐fed rats should not be considered direct evidence of metabolic improvement. Rather, it should be interpreted together with the reductions in body weight, visceral and epididymal fat mass, and terminal lipid concentrations observed in the HPD groups. Differences at lower taxonomic levels provide a more informative basis for interpreting the microbial response. *Allobaculum* was enriched in HPD1 and HPD2, whereas *Lactobacillus* and *Turicibacter* reached their highest relative abundances in HPD2. Previous experimental work has associated changes in *Allobaculum* abundance with diet‐dependent regulation of intestinal lipid handling and metabolic responses; however, the direction and significance of this association vary across models and cannot be established from relative‐abundance data alone (Zheng et al. 2021). Similarly, supplementation with specific *Lactobacillus acidophilus* strains has been reported to modify gut microbiota composition and reduce fat accumulation in high‐fat‐diet‐induced obese mice (Zhang et al. 2024). In the present experiment, however, HPD2 did not show greater body‐weight loss or lower regional fat mass than HPD1. Consequently, the increased relative abundance of *Lactobacillus* in HPD2 is best interpreted as evidence of probiotic‐associated microbial modulation rather than proof of an additional anti‐obesity effect. The increased abundance of *Turicibacter* in HPD2 is particularly noteworthy because this genus has recently been linked to bile‐acid and lipid metabolism. Lynch et al. (2023) demonstrated that different *Turicibacter* strains possess bile salt hydrolase activities that modify host bile‐acid profiles in a strain‐specific manner; expression of *Turicibacter*‐derived bile‐modifying genes in colonizing bacteria was also associated with lower serum cholesterol, triglycerides and adipose tissue mass in gnotobiotic mice. In the present study, the higher abundance of *Turicibacter* in HPD2 may therefore represent a microbial signature potentially relevant to lipid metabolism. Nevertheless, because bile‐acid profiles and bile salt hydrolase activity were not measured, this proposed link remains hypothetical rather than experimentally demonstrated. The observed differences in Bacteroidaceae, Prevotellaceae, Eubacteriaceae and Clostridiaceae may also be relevant to intestinal fermentation. Members of these bacterial families include organisms capable of generating short‐chain fatty acids (SCFAs), including acetate, propionate and butyrate, from fermentable dietary substrates. SCFAs have been implicated in the regulation of intestinal barrier integrity, inflammatory tone and adipose tissue lipid metabolism (May and den Hartigh 2021; Jian et al. 2022). However, protein‐rich diets may also promote the formation of nitrogenous fermentation products when undigested protein reaches the colon, and the metabolic consequences depend on the protein source, digestibility and overall diet composition (Bartlett and Kleiner 2022; Wu et al. 2022). Therefore, the enrichment of potentially fermentative taxa in HPD2 is compatible with altered microbial metabolic activity, but it cannot be equated with increased production of beneficial SCFAs without direct metabolite measurements.

## 5 Conclusion

The present study indicates that a whey protein‐based high‐protein diet was the principal factor associated with improved body‐weight and adiposity outcomes in diet‐induced obese Wistar rats. Over the 12‐week intervention period, high‐protein feeding was associated with reduced food intake, sustained body‐weight loss, and lower visceral and epididymal fat masses. It was also associated with favorable terminal triglyceride and total cholesterol concentrations compared with the unsupplemented normal‐protein diet, while fasting glycemia remained unchanged across experimental groups. Under high‐protein conditions, *Lactobacillus acidophilus* supplementation did not produce a detectable additional reduction in body weight, regional fat mass or serum lipid concentrations. Its contribution was more evident through modulation of gut microbiota composition, including differences in bacterial taxa potentially relevant to intestinal fermentation and lipid‐related metabolic interactions. Nevertheless, because microbial metabolites and functional pathways were not directly assessed, these compositional changes should be interpreted as associative rather than mechanistically confirmed. High‐protein feeding was accompanied by higher terminal urea and uric acid concentrations, whereas creatinine did not show a progressive increase during the intervention. This profile may reflect adaptation to increased dietary nitrogen intake; however, serum markers alone are insufficient to establish renal safety or toxicity. Overall, these findings support whey protein‐based high‐protein feeding as the primary nutritional intervention associated with reduced adiposity and improved lipid‐related outcomes in this obesity model. *L. acidophilus* may offer a complementary microbiota‐modulating contribution, but its additional metabolic benefit requires confirmation through studies integrating microbial metabolite profiling, renal functional assessment and histopathological evaluation.

## Author Contributions

**Aicha Sbaihia:** conceptualization, writing – original draft, validation, formal analysis. **Temesgen Anjulo Ageru:** writing – review and editing, writing – original draft, visualization, supervision, formal analysis. **Wasim S. M. Qadi:** visualization, validation, methodology, formal analysis, investigation. **Bouasria Benbouziane:** investigation, methodology, validation, formal analysis, data curation. **Ahmed Mediani:** writing – review and editing, investigation, project administration, supervision, data curation, resources. **Soumia Keddari:** validation, investigation, software, resources, project administration. **Muhammad Waqar:** conceptualization, visualization, writing – original draft. **Mohamed Cherif Bentahar:** investigation, validation, methodology, software, formal analysis. **Said Dahmouni:** conceptualization, investigation, validation, formal analysis, software. **Djilali Benabdelmoumene:** supervision, writing – review and editing, validation, conceptualization, investigation, resources.

## Funding

The authors have nothing to report.

## Ethics Statement

The animal research presented in this manuscript was carried out ethically in accordance with the Helsinki Declaration and the ARRIVE guidelines for in vivo experiments. Furthermore, the Ethics Committee of the Faculty of Life Science and Nature, affiliated with Abdelhamid Ibn Badis University of Mostaganem, approved this research (Approval No. 2019‐013).

## Conflicts of Interest

The authors declare no conflicts of interest.

## Data Availability

The data are available from the corresponding author upon reasonable request.
